# The complete chloroplast genome of *Galega officinalis* L.

**DOI:** 10.1080/23802359.2021.1878961

**Published:** 2021-03-01

**Authors:** Wenxuan Du, Wenbo Jiang, Dengxia Yi, Yongzhen Pang

**Affiliations:** Institute of Animal Sciences, Chinese Academy of Agricultural Sciences, Beijing, China

**Keywords:** Chloroplast genome, *Galega officinalis* L., phylogenetic analysis

## Abstract

*Galega officinalis* L. is a perennial herb of the Fabaceae family. The flowers of *G. officinalis* L. are colorful and suitable for ornamental purposes. It can be used as a food complement for animals and humans, and it could promote lactation in animals and humans. In this study, we obtained the complete chloroplast genome of *G. officinalis* L. and found it is 125,086 bp in length. The GC content of this genome is 34.18%. Among the 112 unique genes in the chloroplast genome of *G. officinalis* L., 30 tRNA, 4 rRNA and 78 protein-coding genes were successfully annotated. We constructed the maximum likelihood (ML) tree with 26 species, and concluded that *G. officinalis* is phylogenetically closely related to the genus of *Cicer*, *Glycine* and *Desmodium.*

*Galega officinalis* L. is a leguminous perennial herb, which is native to southern Europe and southwest Asia (Oubre et al. 1997). It can be used as animal feed, herbal medicine and ornamental plant, and also used to treat diabetes during medieval times (Atanasov and Tchorbanov 2002). In particular, *G. officinalis* L. can increase lactation in humans and animals (Leporatti and Ivancheva 2003). However, few studies have focused on the molecular aspects of *G. officinalis* L.

Chloroplast is an important organelle with its own genome. Studies on chloroplast genome has been a hot topic in plant molecular evolution and systematics (Clegg et al. 1994). Several features of chloroplast genome have facilitated molecular evolutionary analyses. However, the chloroplast genome sequences of *G. officinalis* L. have not been reported so far. In the present study, the chloroplast genome of *G. officinalis* L. was sequenced and its structural features were characterized, which will be a valuable resource for in-depth studies of the genetic evolution within the Fabaceae family.

Seeds of *G. officinalis* L. were kept at the Forage Germplasm Bank at Institute of Animal Sciences of the Chinese Academy of Agricultural Sciences (IAS-CAAS) (Beijing, E116°29′, N40°03′). The voucher specimen (FR004) was deposited at the Herbarium of the Department of Grassland, IAS-CAAS, Beijing, China. After germinating in the laboratory, genomic DNA from young leaves was extracted using a DNA Extraction Kit from Tiangen Bio Tech Co., Ltd (Beijing, China). The sequencing was carried out on the Illumina Novaseq PE150 platform (Illumina Inc, San Diego), and 150 bp paired-end reads were generated. The software GetOrganelle v1.5 (Jin et al. 2019) was used to assemble the cleaned reads into a complete chloroplast genome, with the chloroplast genome of *Medicago truncatula* (GenBank accession number: NC_003119) used as a reference. The chloroplast genome annotation was performed through the online program CPGAVAS2 (Shi et al. 2019) and GeSeq (Tillich et al. 2017), followed by manual correction. The assembled chloroplast genome sequence with annotation was submitted to GenBank database under the accession number MT506239.

In the present study, we found the complete chloroplast genome of *G. officinalis* L. is 125,086 bp in length, which is a typical circular structure. The GC content of its genome is 34.18%. The chloroplast genome has in total 112 genes, including 78 protein-coding genes, 30 tRNA genes, and 4 rRNA genes. Among them, 30 genes encoding amino acid transfer protein, 21 genes encoding ribosomal structural proteins protein, 17 genes encoding electron transport protein, 14 genes encoding light collection structural protein (PSII) are found in the chloroplast genome of *G. officinalis* L. (Table 1).

**Table 1.** Gene predicted of the chloroplast genome of *Galega officinalis* L.

Biological function	Gene list	Gene number
ATP synthase related	*atpA*, *atpF*, *atpH*, *atpI*, *atpE*, *atpB*	6
ATP-dependent Clp protease	*clpP*	1
Carbon fixation	*rbcL*	1
Fatty acid biosynthesis	*accD*	1
Nucleotide metabolism	*rpoA*, *rpoC2*, *rpoC1*, *rpoB*	4
Photosynthesis: electron transport	*ndhF*, *ndhD*, *ndhE*, *ndhG*, *ndhI*, *ndhA*, *ndhH*, *ndhB*, *petD*, *petB*, *petG*, *petL*, *petA*, *petN*, *ndhJ*, *ndhK*, *ndhC*	17
Photosynthesis: light collection structural protein (PSI)	*psaC*, *psaJ*, *psaI*, *psaB*, *psaA*	5
Photosynthesis: light collection structural protein (PSII)	*psbA*, *psbH*, *psbT*, *psbB*, *psbE*, *psbF*, *psbL*, *psbJ*, *psbK*, *psbI*, *psbM*, *psbD*, *psbC*, *psbZ*	14
Photosynthesis related	*ccsA*, *pbf1*	2
Possible PSI structural protein	*ycf4*, *ycf3*	2
Protein synthesis: amino acid transfer	*trnL-UAG*, *trnN-GUU*, *trnR-ACG*, *trnA-UGC*, *trnE-UUC*, *trnV-GAC*, *trnL-CAA*, *trnM-CAU*, *trnP-UGG*, *trnW-CCA*, *trnQ-UUG*, *trnS-GCU*, *trnR-UCU*, *trnC-GCA*, *trnD-GUC*, *trnY-GUA*, *trnE-UUC*, *trnT-GGU*, *trnS-UGA*, *trnG-GCC*, *trnM-CAU*, *trnS-GGA*, *trnT-UGU*, *trnL-UAA*, *trnF-GAA*, *trnM-CAU*, *trnK-UUU*, *trnH-GUG*, *trnT-CGU, trnI-AAU*	30
RNA splicing	*matK*	1
Ribosomal structural proteins	*rpl32*, *rps15*, *rps12*, *rps7*, *rpl23*, *rpl2*, *rps19*, *rpl22*, *rps3*, *rpl16*, *rpl14*, *rps8*, *rpl36*, *rps11*, *rpl20*, *rps18*, *rpl33*, *rps16*, *rps2*, *rps14*, *rps4*	21
Ribosomal structural RNAs	*rrn5*, *rrn4.5*, *rrn23*, *rrn16*	4
Envelope membrane protein	*cemA*	1
Unknown	*ycf1*, *ycf2*	2

The chloroplast genomes of 26 species from Fabaceae as well as *Vachellia flava* as outgroup species was downloaded from the NCBI GenBank database to inference the phylogenetic relationship of *G. officinalis* L. The sequences were aligned using MAFFT v7 (Katoh et al. 2019). In addition, a maximum likelihood (ML) tree based on the common protein-coding genes of 26 species was constructed by using IQ-TREE under the model automatically selected by IQ-TREE (Nguyen et al. 2015). Phylogenetic analysis shows that *G. officinalis* L is the sister clade of the clade containing species of *Cicer*, *Glycine* and *Desmodium* (Figure 1). It was noticed that the *Galega* chloroplast genome lacks the inverted repeats (IR) as *Cicer arietinum*, but this atypical plastome architecture can be found in some other clades of the Fabaceae family, including *Glycine max* and *Lotus japonicus* (Wang et al. 2017). Finally, this study will provide important information for species identification, molecular evolutionary and phylogenetic relationship at different taxonomic levels within the Fabaceae family.

**Figure 1.**
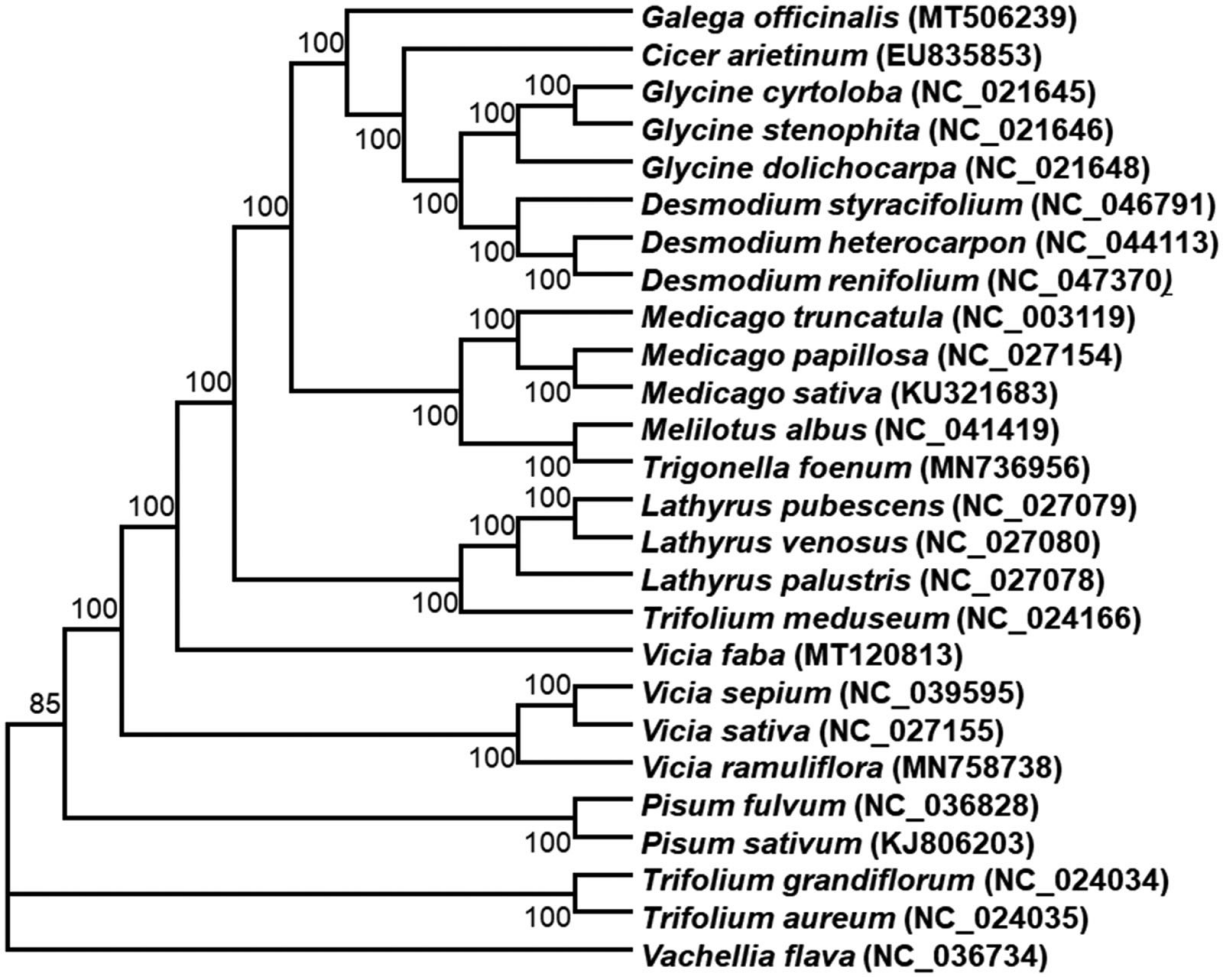
Phylogenetic tree reconstructed using maximum likelihood (ML) method based on the common protein-coding genes of 26 species of the Fabaceae family, with *V. flava* as the outgroup by using IQ-TREE. Numbers above the lines represent ML bootstrap values (>70%).

## Data Availability

The data that support the findings of this study are openly available in NCBI at Genbank with accession number MT506239 (https://www.ncbi.nlm.nih.gov/nuccore/MT506239) Raw sequencing reads used in this study was deposited in the public repository SRA with accession number SRR12678613 (https://www.ncbi.nlm.nih.gov/sra/?term=SRR12678613).
